# Delivery of Phosphorescent Anticancer Iridium(III) Complexes by Polydopamine Nanoparticles for Targeted Combined Photothermal‐Chemotherapy and Thermal/Photoacoustic/Lifetime Imaging

**DOI:** 10.1002/advs.201800581

**Published:** 2018-08-15

**Authors:** Dong‐Yang Zhang, Yue Zheng, Hang Zhang, Jing‐Hua Sun, Cai‐Ping Tan, Liang He, Wei Zhang, Liang‐Nian Ji, Zong‐Wan Mao

**Affiliations:** ^1^ MOE Key Laboratory of Bioinorganic and Synthetic Chemistry School of Chemistry Sun Yat‐Sen University Guangzhou 510275 P. R. China

**Keywords:** combined therapy, iridium complexes, polydopamine, thermal/photoacoustic/lifetime imaging

## Abstract

Recently, phosphorescent iridium complexes have demonstrated great potential as anticancer and imaging agents. Dopamine is a melanin‐like mimic of mussel adhesive protein that can self‐polymerize to form polydopamine (PDA) nanoparticles that demonstrate favorable biocompatibility, near‐infrared absorption, and photothermal effects. Herein, PDA nanoparticles are functionalized with β‐cyclodextrin (CD) substitutions, which are further assembled with adamantane‐modified arginine‐glycine‐aspartic acid (Ad‐RGD) tripeptides to target integrin‐rich tumor cells. The thus formed PDA‐CD‐RGD nanoparticles can deliver a phosphorescent iridium(III) complexes LysoIr ([Ir(ppy)_2_(l)]PF_6_, ppy = 2‐phenylpyridine, L = (1‐(2‐quinolinyl)‐β‐carboline) to form a theranostic platform LysoIr@PDA‐CD‐RGD. It is demonstrated that LysoIr@PDA‐CD‐RGD can be applied for targeted combined cancer photothermal‐chemotherapy and thermal/photoacoustic/two‐photon phosphorescence lifetime imaging under both in vitro and in vivo conditions. This work provides a useful strategy to construct multifunctional nanocomposites for the optimization of metal‐based anticancer agents for further biomedical applications.

## 1 Introduction

Iridium complexes are considered to be promising alternatives to platinum‐based metallo‐anticancer drugs.^1^ Meanwhile, cyclometalated iridium(III) complexes have gained increasing attention in bioimaging and biosensing applications attributed to their rich photophysical properties, e.g., high quantum yields, large Stokes shifts, long emission lifetimes, and good photostability.^2^ However, the clinical applications of iridium complexes are hindered by limited water solubility and poor tumor‐targeting capability. Delivery of anticancer agents by nanoparticles may optimize their bioavailability, intracellular uptake efficacy, and tumor‐targeting capability.^3^ At the same time, nanoparticles can act as multimodal imaging agents with therapeutic capacity, e.g., photothermal therapy and radiotherapy.^4^ The combination of anticancer agents with delivery nanomaterials can achieve synergy effects and provides a novel theranostic approach for simultaneous cancer diagnosis and treatment.^5^

Two‐photon absorption (TPA) materials that can be excited by near‐infrared (NIR) light (700–1100 nm) have decreased photobleaching effects, minimal photodamage of cellular structures, low background interference, and long penetration depth (>500 nm).^6^ Thus, two‐photon microscopy becomes an attractive tool to visualize biological events within live cells and intact tissues. In recent years, emerging studies show that iridium(III) complexes are good candidates as TPA materials and luminophores.[[qv: 2c,7]] On the other hand, fluorescence lifetimes are sensitive to internal factors affecting the structure of the fluorophore, and external factors including temperature, polarity, and the presence of fluorescence quenchers.[[qv: 9a]] The lifetime of a fluorophore does not depend on its concentration, excitation wavelength and duration of light exposure.^8^ Moreover, it is not affected by photobleaching. All these features make lifetime‐resolved imaging an accurate and reliable method to measure the microenvironmental changes of a fluorophore.^9^ Owing to their high spin–orbit coupling associated with the heavy metal ion, metal complexes are ideal candidates for phosphorescence lifetime imaging microscopy (PLIM) with emission lifetimes up to microsecond that can totally deplete the background emission (usually below 10 ns).[[qv: 2e,10]] Thus, two‐photon phosphorescence lifetime imaging (TP‐PLIM) can reflect the microenvironments of metal complexes with high sensitivity and accuracy.[[qv: 10a,c–e]]

Dopamine is a small‐molecule mimic of the adhesive proteins of mussels. DA can self‐polymerize at alkaline pH values to generate polydopamine (PDA) nanoparticles.^11^ Stable PDA nanoparticles can be easily produced with a simple and cost‐effective strategy and are completely composed of naturally occurring dopamine, and they have high biocompatibility and are suitable for biomedical applications.^12^ Moreover, the functional groups (i.e., catechol and amine) on the surface of PDA nanoparticles are available for further functionalization with targeting/recognition elements.[[qv: 11c]] Moreover, imaging‐guided photothermal therapy (PTT) is emerging as a novel therapeutic method for precision therapy that can effectively eliminate cancer cells with precise guidance.^13^ PDA nanoparticles exhibit excellent photothermal conversion efficiency under NIR light irradiation and are emerging as a new kind of photothermal therapeutic agents.[[qv: 12d]]

**Scheme 1:**
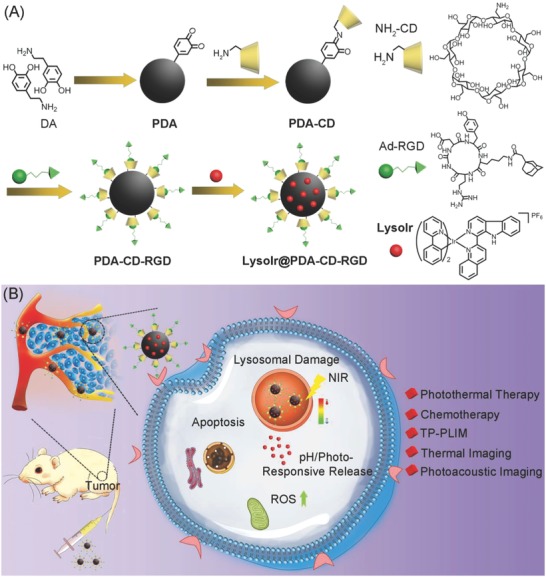
Schematic illustration of A) the synthetic procedures and B) action mechanisms of LysoIr@PDA‐CD‐RGD.

In this work, [Ir(ppy)_2_(L)]PF_6_ (LysoIr, ppy = 2‐phenylpyridine, L = (1‐(2‐quinolinyl)‐β‐carboline), a phosphorescent iridium(III) complex with high TPA properties and potent anticancer activity developed by our group, is used as the chemotherapeutic agent as well as the TP‐PLIM agent.[[qv: 2c]] PDA nanoparticles are modified with β‐cyclodextrin (CD) and self‐assembled with adamantane‐modified arginine‐glycine‐aspartic acid (Ad‐RGD) tripeptides to form PDA‐CD‐RGD. PDA‐CD‐RGD nanoparticles can deliver LysoIr to integrin‐rich tumor cells for combined photothermal‐chemotherapy and photothermal/photoacoustic/TP‐PLIM imaging (**Scheme** **1**). The structure, composition, photothermal conversion capacity, drug loading and release properties of LysoIr@PDA‐CD‐RGD nanoparticles are characterized. The cellular uptake and localization, the combined chemotherapy/photothermal effects and cell death mechanisms are investigated in cell‐based assays. Finally, the tumor and organ distribution/metabolism, the photothermal/photoacoustic imaging capabilities and the combined photothermal‐chemotherapy treatment efficacy of LysoIr@PDA‐CD‐RGD nanoparticles were investigated in vivo. We demonstrate that the easily fabricated and modified nanocomposite can achieve high tumor accumulation through active and passive mechanisms, and it holds high potential in multimodal imaging‐guided combination cancer therapy.

## 2 Results and Discussion

### 2.1 Synthesis and Characterization of LysoIr@PDA‐CD‐RGD

The synthetic procedures of LysoIr@PDA‐CD‐RGD nanoparticles are depicted in Scheme 1A. First, PDA nanoparticles were synthesized by self‐polymerization of dopamine under alkaline conditions (pH = 8.5) with oxygen as the oxidant in a mixture of de‐ionized water and iso‐propyl alcohol (v/v, 5:1) according to literature methods.[[qv: 12d]] Then, mon‐6‐amino‐6‐deoxygen‐β‐cyclodextrin (NH_2_‐CD) was conjugated to PDA via condensation of the amine groups on NH_2_‐CD with the dihydroxyindole/indolequinone groups on PDA to form PDA‐CD. LysoIr was synthesized according to the published methods[[qv: 2c]] and loaded onto PDA‐CD. Finally, Ad‐RGD was absorbed onto LysoIr@PDA‐CD via host‐guest interaction between the adamantane group and the β‐cyclodextrin moiety to form LysoIr@PDA‐CD‐RGD.

Fourier transform infrared (FT‐IR) spectrum (Figure S1, Supporting Information) shows the typical peaks of the benzene ring and the hydroxy group in PDA at 1494 and 3200 cm^−1^, respectively. The successful modification of PDA‐CD is indicated by the peaks at 1031 and 1156 cm^−1^ that can be assigned to the vibrational bands of C—O and C—C bonds in NH_2_‐CD moieties, respectively.

**Figure 1:**
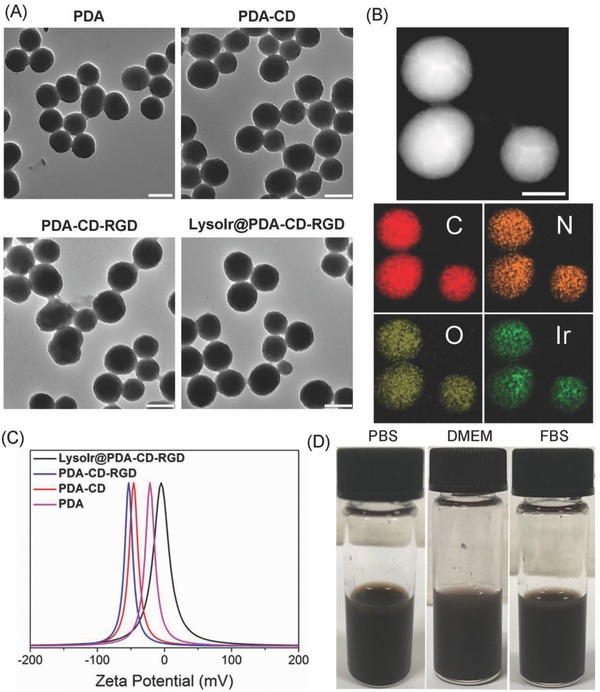
A) TEM images of PDA, PDA‐CD, PDA‐CD‐RGD, and LysoIr@PDA‐CD‐RGD. Scale bar: 200 nm. B) TEM image and EDS elemental maps of LysoIr@PDA‐CD‐RGD. Scale bar: 100 nm. C) Zeta potentials of PDA, PDA‐CD, PDA‐CD‐RGD, and LysoIr@PDA‐CD‐RGD. D) The images of LysoIr@PDA‐CD‐RGD dissolved in PBS, DMEM, and FBS.

Transmission electron microscopy (TEM) (**Figure** **1**A) reveals that the nanoparticles are uniform and well‐dispersed. The average diameters of PDA, PDA‐CD, PDA‐CD‐RGD, and LysoIr@PDA‐CD‐RGD are all about 150 nm. Dynamic light scattering (DLS) measurement also demonstrates that no aggregation occurs (Figure S2, Supporting Information). The average hydrodynamic diameters of PDA and LysoIr@PDA‐CD‐RGD nanoparticles are ≈170 and 222 nm, respectively.

TEM in conjunction with energy‐dispersive X‐ray spectroscopy (TEM‐EDS) elemental maps of the nanoparticles were recorded to identify the element distribution of LysoIr@PDA‐CD‐RGD. Four elements (C, N, O, and Ir) are identified in LysoIr@PDA‐CD‐RGD (Figure 1B; Figure S3, Supporting Information). The distribution map indicates that LysoIr is loaded onto the surface of PDA‐CD‐RGD possibly via π–π interaction as well as intermolecular hydrophobic interaction. Meanwhile, the zeta potentials of PDA, PDA‐CD, PDA‐CD‐RGD, and LysoIr@PDA‐CD‐RGD are measured to be −22, −47, −53, and −5 mV, respectively (Figure 1C), which confirms the conjugation of the negatively charged NH_2_‐CD, RGD, and the subsequent absorption of the positively charged LysoIr. The LysoIr@PDA‐CD‐RGD nanoparticles are well dispersed and exhibit excellent stability in biological mediums for several days, including phosphate buffered saline (PBS), cell medium (Dulbecco's modified eagle medium, DMEM), and fetal bovine serum (FBS) (Figure 1D). The average hydrodynamic diameters of PDA and LysoIr@PDA‐CD‐RGD NPs show barely no change in water for 72 h, which indicates they possess high aqueous stability (Figure S2, Supporting Information).

### 2.2 Drug Loading and Stimulation‐Responsive Release Properties

**Figure 2:**
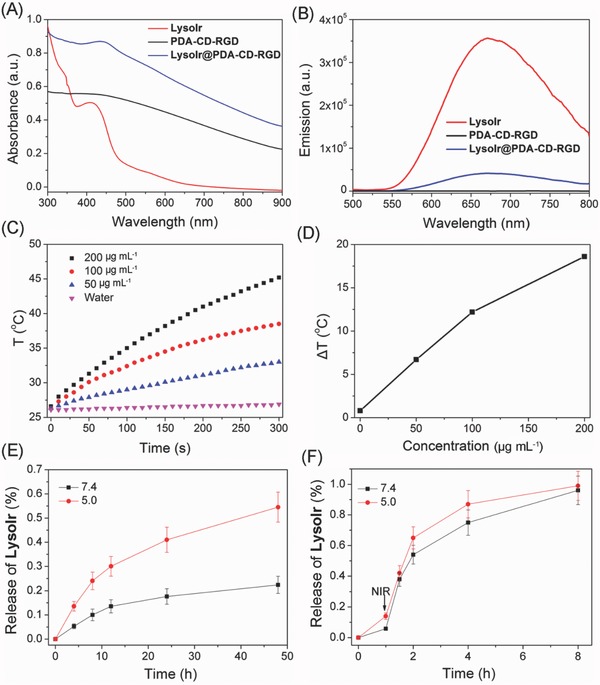
A) UV/vis spectra of PDA‐CD‐RGD, LysoIr, and LysoIr@PDA‐CD‐RGD. B) Fluorescence spectra of PDA‐CD‐RGD, LysoIr, and LysoIr@PDA‐CD‐RGD upon excitation at 405 nm. C) The photothermal profiles of pure water and aqueous dispersions of LysoIr@PDA‐CD‐RGD nanoparticles at different concentrations under 808 nm laser irradiation (1 W cm^−2^). D) Plot of temperature change (Δ*T*) of LysoIr@PDA‐CD‐RGD nanoparticles solution at different concentrations over a period of 5 min. E) In vitro pH‐dependent release of LysoIr from LysoIr@PDA‐CD‐RGD. F) In vitro photothermal‐triggered release of LysoIr from LysoIr@PDA‐CD‐RGD. The samples were irradiated with an 808 nm laser irradiation (1 W cm^−2^) at 1 h.

The UV/vis absorption spectrum of LysoIr@PDA‐CD‐RGD clearly shows the characteristic absorption band of LysoIr with a maximum at ≈418 nm (**Figure** **2**A). The peak at 418 nm was then used to determine the concentrations of LysoIr in LysoIr@PDA‐CD‐RGD. The absorbance contributed by PDA‐CD‐RGD was subtracted. The loading capacity (the weight ratio of LysoIr in LysoIr@PDA‐CD‐RGD) is calculated to be about 5%. Fluorescence spectra show that the emission intensity of LysoIr@PDA‐CD‐RGD is diminished as compared with that of LysoIr (Figure 2B). The quenching effects may be caused by the photo‐induced electron transfer from LysoIr to PDA, which has also been reported for other chromophores absorbed on PDA.[[qv: 11c,12d]]

After loading of LysoIr, LysoIr@PDA‐CD‐RGD nanoparticles maintain a high absorption in the NIR region as PDA (Figure 2A). In order to further prove the potential of LysoIr@PDA‐CD‐RGD as a PTT agent, LysoIr@PDA‐CD‐RGD solutions were exposed to an 808 nm NIR laser (1 W cm^−2^) for different time intervals. Upon irradiation, the temperature of LysoIr@PDA‐CD‐RGD and PDA solutions rapidly increases, while the temperature of pure water barely changes (Figure 2C,D; Figure S4, Supporting Information). The photothermal efficacies of PDA and LysoIr@PDA‐CD‐RGD are similar. After an irradiation for 5 min, the temperature of the LysoIr@PDA‐CD‐RGD solution at a concentration of 200 µg mL^−1^ increases from 26 °C to about 45 °C.

In vitro release of LysoIr from LysoIr@PDA‐CD‐RGD was investigated in PBS by UV/vis spectroscopy. LysoIr@PDA‐CD‐RGD is rather stable at physiological pH (Figure 2E). The release of LysoIr from LysoIr@PDA‐CD‐RGD is accelerated in the acidic solution (pH 5.0) mimicking the lysosomal/endosomal environments. After an incubation for 48 h at 25 °C, about 48% of LysoIr is released from LysoIr@PDA‐CD‐RGD at pH 5.0. Interestingly, as LysoIr is absorbed on LysoIr@PDA‐CD‐RGD via noncovalent interactions, LysoIr can also be released from PDA by photothermal heating (Figure 2F). The irradiation treatment was performed with an 808 nm laser (1 W cm^−2^) at 1 h. Almost all of LysoIr is released from LysoIr@PDA‐CD‐RGD after a further incubation for 7 h.

### 2.3 In Vitro Combined Photothermal‐Chemotherapy Activities

**Figure 3:**
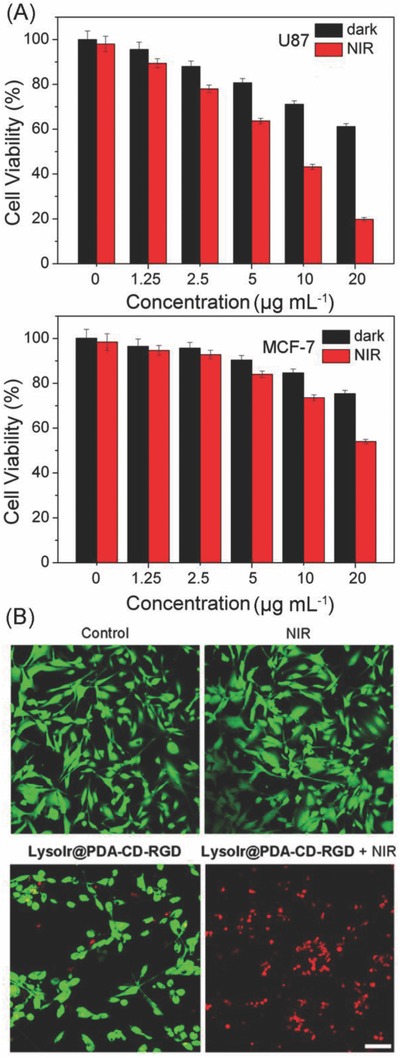
A) Relative viability of U87 and MCF‐7 cells incubated with various concentrations of LysoIr@PDA‐CD‐RGD in the presence and absence of an 808 laser irradiation (1 W cm^−2^, 10 min). B) Calcein AM and PI staining of U87 cells treated with LysoIr@PDA‐CD‐RGD (25 µg mL^−1^, 8 h) and irradiated with an 808 laser (1 W cm^−2^, 10 min). Cells were imaged by a confocal microscope. Calcein AM: λ_ex_ = 488 nm, λ_em_ = 520 ± 20 nm; PI: λ_ex_ = 514 nm, λ_em_ = 640 ± 20 nm. Scale bar: 30 µm.

In vitro combined photothermal‐chemotherapy efficacy of LysoIr@PDA‐CD‐RGD was determined in both integrin‐positive human brain glioma U87 cells, integrin‐negative human breast cancer MCF‐7 cells, human normal liver LO2 cells and human normal breast epithelial MCF‐10A cells by 3‐(4,5‐dimethylthiazol‐2‐yl)‐2,5‐diphenyltetrazolium bromide (MTT) assay. PDA and PDA‐CD nanoparticles display negligible cytotoxicity in the dark (Figure S5, Supporting Information). Combined treatment causes significantly higher cytotoxicity than chemotherapy alone in U87 cells (**Figure** **3**A). After treatment with LysoIr@PDA‐CD‐RGD at 20 µg mL^−1^, the cell viabilities for chemotherapy and combined treatment are 61.2 ± 1.3 and 19.8% ± 0.8%, respectively. The results indicate that the combination with PTT treatment can greatly enhance the anticancer effect of chemotherapy. The death‐inducing capability of LysoIr@PDA‐CD‐RGD in MCF‐7 cells is much lower than that observed in U87 cells under the same conditions (Figure 3A), which indicates that LysoIr@PDA‐CD‐RGD can selectively target integrin‐positive cancer cells. Moreover, the in vitro safety evaluation of LysoIr@PDA‐CD‐RGD in LO2 and MCF‐10A cell lines indicates that the NPs possess low toxicity against these normal cells either in dark or under NIR light irradiation (Figure S6, Supporting Information).

In addition, live/dead cell staining was used to visualize cell viability directly by fluorescence microscope. Live and dead cells were stained with 3′,6′‐di(O‐acetyl)‐4′,5′‐bis[*N*,*N*‐bis(carboxymethyl)aminomethyl]fluorescein (Calcein‐AM) and propidium iodide (PI), respectively. As shown in Figure 3B, compared with the control group, the proliferation of U87 cells treated with LysoIr@PDA‐CD‐RGD is greatly inhibited. An obvious increase in the percentage of dead cells (PI‐positive) is observed in cells treated with LysoIr@PDA‐CD‐RGD and irradiated with an 808 nm laser.

### 2.4 Cellular Uptake and Localization of LysoIr@PDA‐CD‐RGD

The cellular uptake of LysoIr@PDA‐CD‐RGD was investigated by inductively coupled plasma‐mass spectrometry (ICP‐MS). As expected, after the cells are treated with LysoIr@PDA‐CD‐RGD (2.5 × 10^−6^ m based on the concentration of LysoIr) for 6 h, the intracellular iridium content measured in U87 cells (224 ± 28 ng per 10^6^ cells) is much higher than that obtained in MCF‐7 cells (20 ± 4.8 ng per 10^6^ cells).

**Figure 4:**
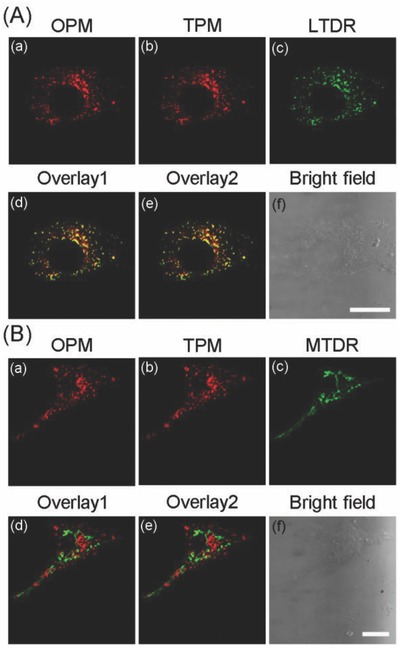
U87 cells were colabeled with LysoIr@PDA‐CD‐RGD (1 × 10^−6^ m based on the concentration of LysoIr, 1 h) and A) LTDR (150 × 10^−9^ m , 0.5 h) or B) MTDR (50 × 10^−9^ m, 0.5 h). MTDR/LTDR: λ_ex_ = 633 nm, λ_em_ = 665 ± 20 nm; OPM (one‐photon microscopy): λ_ex_ = 405 nm, λ_em_ = 680 ± 20 nm; TPM (two‐photon microscopy): λ_ex_ = 810 nm, λ_em_ = 680 ± 20 nm. (d) Overlay of (a) and (c). (e) Overlay of (b) and (c). Scale bars: 10 µm.

High colocalization is observed for LysoIr@PDA‐CD‐RGD with the lysosomal specific dye LysoTracker Deep Red (LTDR) under both one‐photon and two‐photon microscopy (**Figure** **4**A). Meanwhile, minimal colocalization of LysoIr@PDA‐CD‐RGD with MitoTracker Deep Red (MTDR) is observed (Figure 4B). The pearson's colocalization coefficients obtained for LysoIr@PDA‐CD‐RGD with LTDR and MTDR are 82% ± 3% and 28% ± 2%, respectively. The result demonstrates that LysoIr@PDA‐CD‐RGD can specifically image lysosomes.

### 2.5 Cell Death Mechanisms Induced by LysoIr@PDA‐CD‐RGD

Based on morphological and biochemical criteria, cell death can be mainly divided into necrosis, apoptosis, and autophagy.^14^ First, the changes in cell morphology of U87 cells treated with LysoIr@PDA‐CD‐RGD were examined by 2′‐(4‐ethoxyphenyl)‐5‐(4‐methyl‐1‐piperazinyl)‐2,5′‐bi‐1H‐benzimidazole trihydrochloride (Hoechst 33342) staining. Control cells show normal overall morphology with nuclei homogeneously stained (Figure S7, Supporting Information). After combined treatment, U87 cells show typical morphological alterations of apoptosis, including cell shrinkage, membrane bubbling, bright staining, condensed chromatin, fragmented nuclei, and apoptotic bodies.^15^ Compared with chemotherapy treatment alone, combined therapy can markedly increase the proportion of cells with abnormal morphology. As LysoIr mainly induces autophagic cell death,[[qv: 2c]] the result suggests that combined treatment of LysoIr with nanomaterials can change the modes of cell death.

**Figure 5:**
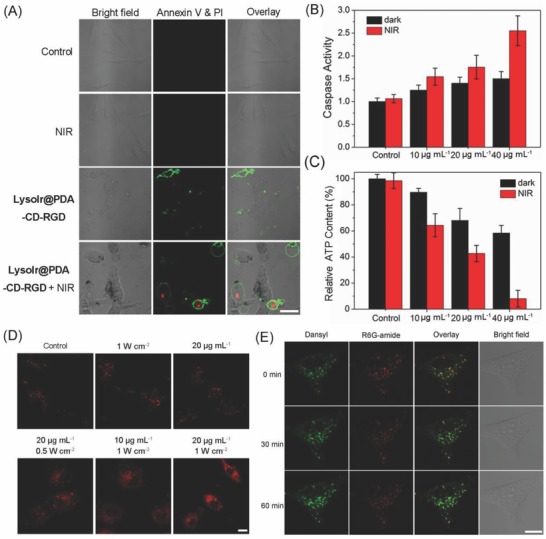
A) Representative confocal microscopic images of annexin V‐FITC/PI double stained U87 cells treated with LysoIr@PDA‐CD‐RGD (20 µg mL^−1^, 12 h) in the absence and presence of light. FITC: λ_ex_ = 488 nm; λ_em_ = 530 ± 20 nm; PI: λ_ex_ = 514 nm; λ_em_ = 620 ± 20 nm. B) Detection of caspase 3/7 activity in U87 cells treated with LysoIr@PDA‐CD‐RGD at the indicated concentrations in the absence or presence of light. C) Detection of cellular ATP content. D) Observation of cathepsin B release from lysosomes to cytosol in U87 cells treated with LysoIr@PDA‐CD‐RGD in the absence or presence of the 808 laser irradiation. λ_ex_ = 543 nm; λ_em_ = 630 ± 20 nm. E) Visualization of lysosomal pH in U87 cells. The cells were treated with LysoIr@PDA‐CD‐RGD (20 µg mL^−1^, 4 h) and stained with Lyso‐DR (1 µg mL^−1^, 30 min). After irradiation, the cells were imaged with a confocal microscope at 0, 30 and 60 min. Dansyl: λ_ex_ = 405 nm; λ_em_ = 440 ± 30 nm. R6G‐amide: λ_ex_ = 543 nm; λ_em_ = 590 ± 30 nm. For the light‐treated samples in (A)‒(E), cells were irradiated with an 808 nm laser at a light dose of 1 W cm^−2^ for 5 min. Scale bar: 10 µm.

Membrane asymmetry is lost and phosphatidylserine (PS) translocates to the external leaflet during early apoptosis, which can be detected by annexin V staining.^15^ Late stage apoptotic cells and necrotic cells will be positively stained by PI due to the lost of the membrane integrity. Annexin V‐FITC/PI double staining shows that cells incubated with LysoIr@PDA‐CD‐RGD and irradiated with an 808 nm laser display apparent features of apoptosis, as evidenced by the appearance of green fluorescence on the cell membrane (**Figure** **5**A). The activation of caspases is considered to be one of the key events during apoptosis.^16^ As compared with the control cells in the dark, negligible increase in caspase 3/7 activity is detected in cells treated with NIR light or LysoIr@PDA‐CD‐RGD alone. The combination of LysoIr@PDA‐CD‐RGD (40 µg mL^−1^) with NIR irradiation (808 nm, 1 W cm^−2^) causes an ≈2.5‐fold increase in caspase 3/7 activity (Figure 5B). Moreover, cells pretreated with the pan‐caspase inhibitor (z‐VAD‐FMK, 50 × 10^−6^ m) show a marked increase in cell viability compared with cells treated with LysoIr@PDA‐CD‐RGD alone or combined therapy (Figure S8, Supporting Information). Western blot analysis also confirms the activation of caspase‐3 induced by LysoIr@PDA‐CD‐RGD‐mediated combined treatment in U87 cells (Figure S9, Supporting Information). Apoptosis is closely associated with the decline in cellular ATP levels.^17^ The ATP level in cells after combined treatment (LysoIr@PDA‐CD‐RGD: 40 µg mL^−1^; NIR light: 1 W cm^−2^) is reduced to about 8% as compared with the control cells (Figure 5C).

In many cases, iridium complexes are reported to induce cell death through the elevation of cellular reactive oxygen species (ROS) levels.[[qv: 1b,c]] Photothermal therapy can also induce ROS generation and oxidative stresses in cancer cells during treatment.^18^ The generation of ROS generation in U87 cells was detected by the 2′,7′‐dichlorofluorescin diacetate (DCFH‐DA) assay. DCFH‐DA is cleaved intracellularly by nonspecific esterases to form dichlorofluorescin (DCFH). The non‐fluorescent DCFH can be oxidized by ROS to highly fluorescent dichlorofluorescein (DCF).^19^ A significant increase in DCF fluorescence is observed in U87 cells treated with LysoIr@PDA‐CD‐RGD alone or in combination with NIR irradiation as measured by confocal microscopy (Figure S10, Supporting Information).

Lysosomal membrane permeabilization can cause the release of lysosomal proteases, e.g., cathepsin B, from lysosomes to cytosol, which ultimately leads to cell death.^18^ As LysoIr@PDA‐CD‐RGD could localize to lysosomes, the release of cathepsin B from lysosomes was detected using the Magic Red MR‐(RR)_2_ assay.^20^ Control cells display red dot‐like fluorescence mostly localized in lysosomes. The release of cathepsin B from lysosomes into cytosol can be observed in cells treated LysoIr@PDA‐CD‐RGD and irradiated with NIR irradiation (Figure 5D). The loss of lysosomal integrity caused by NIR light may trigger the effective drug release from lysosomes into the cytosol. The pH changes of lysosomes were further investigated by LysoSensor DR (Lyso‐DR) staining.^21^ After irradiation, an increase in the fluorescence of dansyl along with a decrease in the emission of R6G‐amide can be observed, which indicated the elevation of lysosomal pH (Figure 5E). No obvious changes in lysosomal pH are observed in the control and chemotherapy groups (Figure S11, Supporting Information). The results indicate that LysoIr@PDA‐CD‐RGD can induce caspase‐mediated apoptotic cell death through lysosomal damage and ROS generation upon NIR light irradiation.

### 2.6 TP‐PLIM by LysoIr@PDA‐CD‐RGD

**Figure 6:**
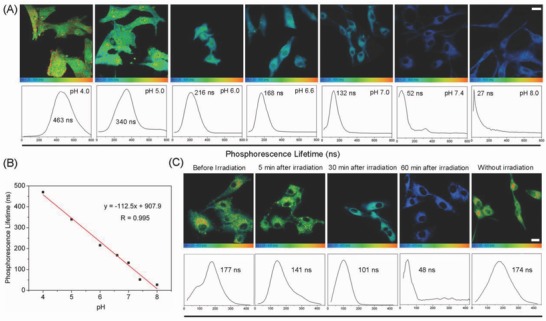
A) TP‐PLIM images of U87 cells incubated with LysoIr (2 × 10^−6^ m, 1 h) at different pH values. λ_ex_ = 810 nm; λ_em_ = 680 ± 20 nm. B) Linear fit between phosphorescence lifetimes of LysoIr and pH values in U87 cells. C) TP‐PLIM images of U87 cells incubated with LysoIr@PDA‐CD‐RGD. The cells were treated with LysoIr@PDA‐CD‐RGD (20 µg mL^−1^, 2 h) and then irradiated with an 808 laser (1 W cm^−2^, 5 min). Scale bar: 10 µm.

Cyclometalated iridium(III) complexes usually display intense emission in the visible region with long lifetimes up to hundreds of nanoseconds.^22^ These complexes have been widely used as steady‐state imaging agents for a variety of small molecules or macrobiomolecules.^23^ We have previously reported that LysoIr can act as a theranostic anticancer agent that can induce autophagy and image lysosomes simultaneously.[[qv: 2c]] Due to the protonation/deprotonation of the secondary amine of the indole rings on the β‐carboline ligands, the emission intensity of LysoIr is dependent on the acidity of the environment. Under neutral and basic conditions, the phosphorescence of LysoIr becomes very weak and “turned‐off.” When the pH is changed from weakly basic (8.0) to acidic (4.0), a significant increase in the emission intensity and lifetime can be observed (Figure S12 and Table S1, Supporting Information).[[qv: 2c]] The possibility of LysoIr to function as a lifetime‐based intracellular pH probe was investigated by adjusting the pH values inside the cells by buffered solutions (**Figure** **6**A). Using TP‐PLIM, the lifetimes imaged under different cellular pH values fall into the range between 27 and 463 ns, which are much longer than the emission lifetimes of organic probes.[[qv: 9a,24]] The emission lifetimes of LysoIr are much longer than those of the cellular endogenous fluorophores (below 10 ns), which indicates that TP‐PLIM can completely eliminate the interference of the background fluorescence. Moreover, a good linear relationship is obtained between the pH values and lifetimes integrated from the images obtained by TP‐PLIM (Figure 6B). These results show that LysoIr can be applied for approximate qualitative measurement of the intracellular pH.

LysoIr@PDA‐CD‐RGD can specifically image lysosomes, and it can also induce lysosomal membrane permeability resulting in the release of the protease and the increase in lysosomal pH upon irradiation.^25^ Therefore, we can use TP‐PLIM to evaluate the degrees of lysosomal damage. The average lifetime of LysoIr measured in U87 cells before NIR light irradiation is about 177 ns (Figure 6C). The average lifetime decreases to about 141 ns 5 min after irradiation. After 60 min, the average lifetime decreases significantly to 48 ns, which indicates the combination therapy causes a significant increase in lysosomal pH. The phenomenon is consistent with the previous results that show the combined treatment can induce lysosomal damage (Figure 5D,E). Measuring intracellular pH has also been achieved with other probes, e.g., quantum dot nanoparticles.^26^ However, the autofluorescence‐free measurement using TP‐PLIM can be more accurate due to the longer lifetime and widened lifetime range. As far as we know, this is the first time that TP‐PLIM is applied to image intracellular pH.

### 2.7 In Vivo Imaging, Biodistribution, and Anticancer Properties of LysoIr@PDA‐CD‐RGD

**Figure 7:**
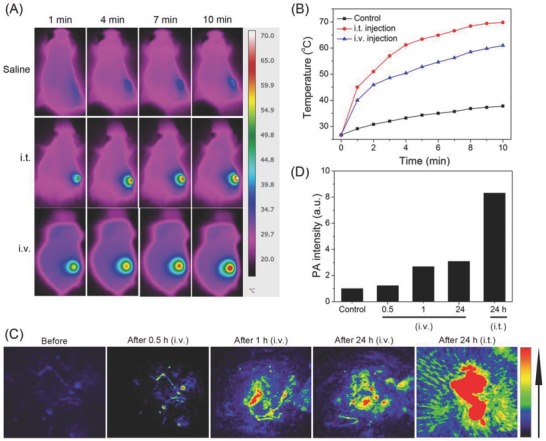
A) Thermal images of U87 tumor‐bearing mice treated with LysoIr@PDA‐CD‐RGD (100 µL, 2 mg mL^−1^, 4 h) and exposed to an 808 nm laser for 10 min (i.t. injected group: 1 W cm^−2^; i.v. injected group: 1.5 W cm^−2^). B) Temperature at the tumor sites monitored by an IR thermal camera at different time points during the irradiation. C) In vivo photoacoustic imaging of tumors in U87 tumor‐bearing mice. The mice were i.v. or i.t. injected with LysoIr@PDA‐CD‐RGD (200 µL, 2 mg mL^−1^). D) The intensity of photoacoustic signals at the tumor sites.

For in vivo thermal imaging of tumors by LysoIr@PDA‐CD‐RGD, an infrared thermal mapping apparatus was used to record the temperature change upon NIR laser irradiation. After 10 min irradiation, a moderate increase in temperature (37.8 °C) is observed in tumors treated with saline (**Figure** **7**A,B). In tumor intratumorally (i.t.) and intravenously (i.v.) injected with LysoIr@PDA‐CD‐RGD (100 µL, 2 mg mL^−1^), the temperature rapidly increases to 69 °C (i.t.) and 61 °C (i.v.), respectively. Photoacoustic imaging tomography (PAT) is a newly emerged biomedical imaging modality that has attracted significant interests in recent years.^27^ PAT imaging is developed based on the photoacoustic effect of light‐absorbers. Compared with traditional *in vivo* optical imaging modalities, e.g., fluorescence imaging, PAT offers remarkably increased imaging depth and spatial resolution.^28^ Mice bearing U87 tumors were i.t. or i.v. injected with LysoIr@PDA‐CD‐RGD (2 mg mL^−1^, 100 µL) and imaged under a PAT imaging system excited with an 810 nm laser. All experimental protocols were approved by the Sun Yat‐Sen University Animal Care and Use Committee. The accreditation number of the laboratory is No.00184195. Strong PA signals in the tumor sites can be detected (Figure 7C,D), which demonstrates that LysoIr@PDA‐CD‐RGD can be effectively delivered to the tumor sites through positive (RGD that recognizes integrin) and passive (enhanced permeability and retention effects of nanoparticles) targeting. Both PAT and photothermal imaging can provide effective tools for imaging‐guided therapy and in situ monitoring of responses to combination therapy.

**Figure 8:**
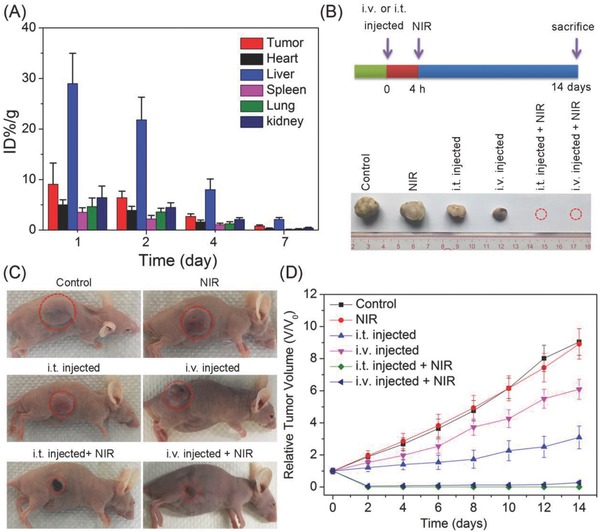
A) Biodistribution of LysoIr@PDA‐CD‐RGD in different organs at various time points after intravenous injection of the nanoparticles. The values were presented as the percentage of injected dose per g of collected organ and based on three mice per group. B) Schematic illustration of LysoIr@PDA‐CD‐RGD‐based combination therapy and representative photos of tumors collected from different groups of mice at the end of treatment. The red dashed circles represent tumors completely disappearing. C) Representative photos of mice after various treatments taken at day 14. Tumor sites are marked with red dashed circles. D) Tumor growth curves of different groups (five mice per group). Error bars were based on standard deviation of mean.

In order to investigate the in vivo biodistribution of LysoIr@PDA‐CD‐RGD, U87 tumor‐bearing Balb/C mice i.v. were injected with LysoIr@PDA‐CD‐RGD (2 mg mL^−1^, 200 µL) and scarified 1, 2, 4, and 7 days post injection. Major organs of mice (*n* = 3) were collected and solubilized by aqua regia for ICP‐MS measurement of iridium content. High levels of Ir element were detected in tumor, as well as reticuloen‐dothelial systems such as liver (**Figure** **8**A). The iridium content in tumor tissue was measured to be about 9.1% ID g^−1^ (the percentage of injected dose per gram tissue) 1 day after injection. After 7 days, the iridium contents in the organs measured decrease to a very low level, indicate that iridium can be effectively removed from the body in a short period of time.

For in vivo the evaluation of anticancer activity, mice bearing U87 tumors with initial volumes of 100–150 mm^3^ were chosen and randomly divided into six groups. For the chemotherapy group and combined photothermal‐chemotherapy group, the mice were i.v. or i.t. injected with LysoIr@PDA‐CD‐RGD. The combined treatment group and the NIR treatment only group were irradiated with an 808 nm laser (i.t. injected group: 1 W cm^−2^; i.v. injected group: 1.5 W cm^−2^; 10 min) 4 h after injection. Tumor volumes and body weights were monitored every 2 days. Mice were sacrificed, and tumors were excised and weighed after treatment for 14 days. A photograph of tumor tissues and a line graph of tumor volumes after various treatments at day 14 show clearly sharp differences in the tumor development among the six groups (Figure 8B‒D). Tumors in the two combination therapy (LysoIr@PDA‐CD‐RGD + NIR laser irradiation) groups are almost completely eradicated without regrowth in 14 days. As compared with the control group in dark, tumor growth is partially delayed in mice injected with LysoIr@PDA‐CD‐RGD in the absence of light. The control group with light treatment shows almost no delay in tumor growth.

Neither obvious body weight loss nor noticeable abnormality is observed for all the groups tested (Figure S13, Supporting Information). Hematoxylin and eosin staining and histology analysis of the major organ reveal no noticeable organ damage or inflammatory lesion in mice subjected to chemotherapy or combined treatment (Figure S14, Supporting Information). These in vivo experiments indicate that LysoIr@PDA‐CD‐RGD holds high potential for imaging‐guided combined photothermal‐chemotherapy.

## 3 Conclusion

In summary, we designed a multifunctional theranostic platform LysoIr@PDA‐CD‐RGD that was formed by integrin‐targeted PDA nanoparticles loaded with an anticancer iridium(III) complex. The successfully fabrication of the nanohybrid was characterized by UV/Vis, FT‐IR, TEM, DLS, TEM‐EDS, and zeta potential measurements. The release of LysoIr from LysoIr@PDA‐CD‐RGD is pH and NIR light dual‐responsive. LysoIr@PDA‐CD‐RGD nanoparticles are selectively in killing U87 cells through combined photothermal‐chemotherapy. In vitro anticancer mechanism investigation indicates that LysoIr@PDA‐CD‐RGD can localize to lysosomes and cause lyososmal damage, ROS elevation, caspase activation and apoptosis in U87 cells. Due to the pH‐dependent phosphorescent lifetime and high TPA of LysoIr, LysoIr@PDA‐CD‐RGD can reflect lysosomal damage in U87 cells using TP‐PLIM technique. Moreover, LysoIr@PDA‐CD‐RGD can image tumor tissues using thermal and photoacoustic methods. Impressively, LysoIr@PDA‐CD‐RGD can eradicate tumor completely and displays barely any side effects. Taken together, we demonstrate the combination of easily synthesized PDA‐based nanomaterials with phosphorescent anticancer iridium(III) complexes have high potential in imaging‐guided anticancer therapy.

## Conflict of Interest

The authors declare no conflict of interest.

## Supporting information

Supplementary
