# Supplementary material for: Delivery of Phosphorescent Anticancer Iridium(III) Complexes by Polydopamine Nanoparticles for Targeted Combined Photothermal‐Chemotherapy and Thermal/Photoacoustic/Lifetime Imaging
Source: Adv Sci (Weinh). 2018 Aug 15;5(10):1800581. doi: 10.1002/advs.201800581 (PMC6193176; doi:10.1002/advs.201800581)
Supplement: Supplementary file 1 — Supplementary [file ADVS-5-1800581-s001.pdf]

Copyright WILEY-VCH Verlag GmbH & Co. KGaA, 69469 Weinheim, Germany, 2018.

ADVANCED SCIENCE
Open Access

Supporting Information

for *Adv. Sci.,* DOI: 10.1002/advs.201800581

Delivery of Phosphorescent Anticancer Iridium(III) Complexes by Polydopamine Nanoparticles for Targeted Combined Photothermal-Chemotherapy and Thermal/ Photoacoustic/Lifetime Imaging

Dong-Yang Zhang, Yue Zheng, Hang Zhang, Jing-Hua Sun, Cai-Ping Tan, Liang He, Wei Zhang, Liang-Nian Ji, and Zong-Wan Mao**

Delivery of Phosphorescent Anticancer Ir(III) Complexes by Polydopamine Nanoparticles for Targeted Combined Photothermal-Chemotherapy and Thermal/Photoacoustic/Lifetime Imaging

Dong-Yang Zhang, Yue Zheng, Hang Zhang, Jing-Hua Sun, Cai-Ping Tan, Liang He, Wei Zhang, Liang-Nian Ji and Zong-Wan Mao**

Electronic Supporting Information

Experimental Section 3
1.1 Materials 3
1.2 General Instruments 3
1.3 Preparation of **LysoIr@PDA-CD-RGD** 4
1.4 Characterization of the Nanoparticles 5
1.5 Photothermal Activity 5
1.6 LysoIr Loading and pH/NIR-Triggered Drug Release 5
1.7 Cell Lines and Culture Conditions 6
1.8 *In Vitro* Cytotoxicity Assay 6
1.9 Calcein AM/PI Staining Assay 7
1.10 Cellular Uptake 7
1.11 Hoechst 33342 Staining 8
1.12 Annexin V/Propidium Iodide Double Staining Assay 8
1.13 Measurement of Caspase 3/7 Activity and Intracellular ATP Levels Assays .9
1.14 Western Blot Analysis 9
1.15 Detection of Intracellular ROS 10
1.16 Detection of Cathepsin B Release 11
1.17 Detection of lysosomal pH 11
1.18 Two-photon Phosphorescence Lifetime Imaging 11
1.19 Animals 12
1.20 *In Vivo* Photothermal and Photoacoustic Imaging 12
1.21 *In Vivo* Biodistribution Measurement of **LysoIr@PDA-CD-RGD** ....12~~13~~
1.22 *In Vivo* Anticancer Properties 13
1.23 Statistical Analysis 14
Supporting Figures and Tables 15
Figure S1 The IR spectra of **PDA**, **PDA-CD** and NH_2-CD 15
Figure S2 The DLS of **PDA** and **LysoIr@PDA-CD-RGD** 15
Figure S3 The EDX spectrum of as-synthesized **LysoIr@PDA-CD-RGD**.........16
Figure S4 The photothermal activity of **PDA** 16
Figure S5 Cytotoxicity of the **PDA** and **PDA-CD**. 17
Figure S6 Cytotoxicity of the **LysoIr@PDA-CD-RGD**. 17
Figure S7 Hoechst 33342 stained assay 18
Figure S8 Caspase inhibitor assay 18
Figure S9 Western blotting assay 18

Figure S10 Detection of cellular ROS levels..19
Figure S11 Visualization of lysosomal pH 20
Figure S12 pH-sensitive phosphorescence lifetime of **LysoIr** 21
Figure S13 Body weight data of mice...21
Figure S14 H&E stained slice...22
Table S1 pH-sensitive phosphorescence lifetime of **LysoIr**.................................22
References..22

1. Experimental Section

1.1 Materials

Iridium trichloride hydrate (J&K Chemical, China), sodium chloride (J&K Chemical, China) NH_2-CD (Zhiyuan Biotechnology Co. Ltd., China), Ad-RGD (cyclic Arg-Gly-Asp peptides (c(RGDyK) conjugated with adamantine, GL Biochem (Shanghai) Ltd., China) were purchased from commercial resouces. Ppy (Sigma Aldrich, USA), dimethyl sulfoxide (DMSO), MTT, disodium hydrogen phosphate, citric acid, chloroquine, DMEM (Dulbecco's modified Eagle's medium), FBS, antibiotics (penicillin/streptomycin) and DCFH-DA were obtained from Sigma-Aldrich (USA). **MTDR** and **LTDR** were purchased from Thermo Fisher Scientific (USA). Calcein AM and PI were purchased from Shanghai Yusheng Biotechnology Co. Ltd. (China), Magic Red MR-$(RR)_2$ was purchased from Immunochemistry Tech (USA). Nigericin was purchased from Cayman Chemical (USA). The Caspase Inhibitor Z-VAD-FMK was purchased from Beyotime Biotechnology. The LysoSensor DR (Blue/Orange) was purchased from Xiamen Bioluminor Bio-Tech Co.,Ltd. All the other chemicals were of analytical grade. Deionized water, purified by a Milli-Q water purification system (Millipore, USA) to a minimum resistivity of 18.2 MΩ cm, was used in all experiments.

1.2 General Instruments

Microanalysis of elements (C, H, and N) was carried out using an Elemental Vario EL CHNS analyzer (Germany). Nuclear magnetic resonance (NMR) spectra were recorded on a Bruker Avance III 400 MHz spectrometer (Germany) at room

temperature. Shifts were referenced relative to the internal solvent signals. Cell imaging experiments were carried out on a confocal microscope (Zeiss LSM-710, ZEISS, Germany).

1.3 Preparation of LysoIr@PDA-CD-RGD

1.3.1 Synthesis of [Ir(ppy)$_2$(L)]PF$_6$ (LysoIr). **LysoIr** was synthesized according to published methods.[1]

1.3.2 Synthesis of Cyclodextrin-modified Polydopamine (PDA-CD). The **PDA** nanoparticles were prepared following a literature procedure.[2] NH_4OH solution (28 wt%) was added to 50 mL of **PDA** aqueous solution (1 mg mL^{-1} in water) to adjust the pH of the solution to 9. The mixture was then added into the aqueous solution (2 mL, pH = 9) of NH_2-CD (50 mg) dropwisely. After vigorous stirring for 12 h, **PDA-CD** was retrieved by centrifugation and washed with deionized water for several times by redispersion/centrifugation processes to remove the unreacted NH_2-CD. The aqueous solvent was removed by freeze-drying and the obtained PDA-CD was weighed to preliminary calculate the quantity of the CD attached on **PDA**.

1.3.3 Preparation of PDA-CD-RGD. Ad-RGD (2 mg) was added into **PDA-CD** dissolved in PBS (1 mL, 20 mg mL^{-1}) and the tube was subjected to ultrasonic for 10 min. Then the tube was shaken for 24 h in the dark. The solid was separated from the solution by centrifuge. The product was washed thrice with PBS and freeze-dried.

1.3.4 Preparation of LysoIr@PDA-CD-RGD. **PDA-CD-RGD** (20 mg) was dispersed in PBS (10 mL) by ultrasonic, and then **LysoIr** (3 mg) dissolved in DMSO

(500 μL) was added. The mixture was stirred for 24 h at room temperature. The solid was separated from the solution by centrifuge and then freeze-dried.

1.4 Characterization of the Nanoparticles

Fourier transform infrared (FT-IR) spectrometry was performed on a Bruker Vector-22 infrared spectrometer (Germany). The morphology of **PDA**, **PDA-CD**, **LysoIr@PDA-CD** and **LysoIr@PDA-CD-RGD** was observed by TEM (JEOL, JEM1400Plus 120 kv, Japan and FEI Tecnai^{TM}, F30 300 kv, USA). NPs were dropped onto a copper grids coated carbon membrane, and dried in the air. DLS and zeta potential were tested by a Zetasizer Nano instrument (EliteSizer, Bruker). TEM in conjunction with energy-dispersive X-ray spectroscopy (TEM-EDS) elemental maps were analyzed by Energy Dispersive X-ray Spectrometer. UV/Vis spectra were recorded on a Varian Cary 300 spectrophotometer (USA). The fluorescence emission spectra were obtained using a Edinburgh FLS 920 Spectrometer (UK).

1.5 Photothermal Activity

PDA and **LysoIr@PDA-CD-RGD** in water were placed in a vial and irradiated with an 808 nm laser (1 W cm^{-2}, 5 min). Light-induced temperature changes in the solutions were recorded using a thermometer sonde. Millipore water was used as the control group.

1.6 LysoIr Loading and pH/NIR-Triggered Drug Release

The concentration of **LysoIr** was quantified by the absorption peak at 418 nm and the absorption of PDA at the same wavelength was subtracted. Fluorescence spectra of free **LysoIr**, **PDA-CD** and **LysoIr@PDA-CD-RGD** were measured upon

excitation at 405 nm. To measure the release of **LysoIr** from the nanohybrid, **LysoIr@PDA-CD-RGD** was incubated in PBS (pH 5.0 or 7.4) and the absorption at 418 nm was measured at different time intervals (0, 4, 8, 12, 24 and 48 h). The amount of **LysoIr** retained on **LysoIr@PDA-CD-RGD** was calculated by absorption at 418 nm after the removal of the detached **LysoIr** by centrifugal filtration. For photothermal-triggered release, the solution of **LysoIr@PDA-CD-RGD** (10 μg mL^{-1}) was irradiated with an 808 nm laser at an output power of 1 W cm^{-2} for 10 min. The irradiation was performed at 1 h. The released **LysoIr** is determined by measuring the UV absorbance at 418 nm of the supernatant separated by centrifugation at different time points.

1.7 Cell Lines and Culture Conditions

MCF-7, MCF-10, LO2 and U87 cells were obtained from Experimental Animal Center of Sun Yat-Sen University (Guangzhou, China). Cells were maintained in DMEM that contained 10% FBS, 100 μg mL^{-1} streptomycin and 100 U/mL penicillin. The cells were cultured in a humidified incubator, which provided an atmosphere of 5% CO_2 and 95% air at a constant temperature of 37 °C.

1.8 *In Vitro* Cytotoxicity Assay

The cells were seeded in 96-well plates and cultured for 24 h. Then the medium was replaced with medium containing different concentrations of **PDA**, **PDA-CD** and **LysoIr@PDA-CD-RGD**. After 24 h, the media was removed and fresh media was added. Then PTT groups were irradiated with an 808 nm laser (1 W cm^{-2}, 10 min). After 20 h, 20 μL MTT (5 mg mL^{-1}) solution was added to each well. The plates were

incubated in the dark for an additional 4 h. The media was carefully removed and DMSO was added (150 μL per well). The plate was incubated at room temprature for 10 min with shaking. The absorbance at 595 nm was measured using a microplate reader (Infinite F200, Tecan, Switzerland). The cells treated under identical conditions in dark were kept as control groups. The percentage of viability was calculated as the following formula: (viable cells)% = (OD of treated sample/OD of untreated sample) × 100%. For the cytotoxicity assay in the presence of the inhibitors, U87 cells were preincubated with 50 μM z-VAD-FMK for 1 h before **LysoIr@PDA-CD-RGD** were added.

1.9 Calcein AM/PI Staining Assay

U87 cells were treated with **LysoIr@PDA-CD-RGD** (25 μg mL^{-1}) for 8 h and irradiated with an 808 nm laser (1 W cm^{-2}, 10 min). After another 4 h, the cells were incubated with calcein AM (2 μM) and PI (5 μM) for 30 min and imaged directly using a confocal microscope. Calcein AM: λ_{ex} = 488 nm; λ_{em} = 520 ± 20 nm. PI: λ_{ex} = 514 nm; λ_{em} = 620 ± 20 nm.

1.10 Cellular Uptake

1.10.1 Colocalization Assay. U87 cells were incubated with **LysoIr@PDA-CD-RGD** (10 μM based on the concentration of **LysoIr**) for 3.5 h and further co-incubated with **LTDR (**50 nM) or **MTDR** (150 nM) at 37 °C for another 30 min. Cells were washed three times with PBS and visualized by confocal microscopy immediately. The one- and two-photon excitation wavelengths of **LysoIr@PDA-CD-RGD** are 405 nm and 810 nm, respectively. The excitation

wavelength of **MTDR**/**LTDR** is 633 nm. Emission was collected at 660 ± 20 nm (**LysoIr**), 665 ± 20 nm (**MTDR**) and 668 ± 20 nm (**LTDR**).

1.10.2 Inductively Coupled Plasma Mass Spectrometry (ICP-MS) Measurement. U87 and MCF-7 cells were seeded in 10 cm tissue culture dishes and incubated for 24 h. The medium was removed and replaced with fresh medium containing **LysoIr@PDA-CD-RGD** (2.5 μM based on the concentration of **LysoIr**). After 6 h incubation, the cells were washed with PBS, trypsinized and collected. The cells were counted, and digested with HNO_3 (65%, 0.2 mL) at room temperature for 24 h. The solution was then diluted to a final volume of 10 mL with Milli-Q water. The concentration of iridium was measured using the XSERIES 2 ICP-MS (Thermo Scientific, USA).

1.11 Hoechst 33342 Staining

U87 cells were seeded into 35 mm dishes (Corning) and incubated for 24 h. The cells were treated with **LysoIr@PDA-CD-RGD** (20 μg mL^{-1}) for 12 h, after which the samples were irradiated with an 808 nm laser (1 W cm^{-2}, 5 min). After incubated for 12 h, the cells were washed twice with PBS and fixed with 4% paraformaldehyde at room temperature for 10 min. Then, the cells were labeled with Hoechst 33342 (5 μg mL^{-1} in PBS) for 5 min and wash twice with PBS. The cells were imaged immediately with a confocal laser-scanning microscope with excitation at 405 nm and emission at 460 ± 20 nm.

1.12 Annexin V/Propidium Iodide Double Staining Assay

The assay was performed according to the manufacturer's (Sigma Aldrich, USA) protocol. U87 cells were treated with **LysoIr@PDA-CD-RGD** (20 μg mL^{-1}) for 12 h. The cells were irradiated with an 808 nm laser (1 W cm^{-2}, 5 min) or not. After 12 h the cells were incubated with 500 μL annexin-binding buffer supplemented with 5 μL annexin V and 10 μL propidium iodide at room temperature for 15 min in the dark. The samples were visualized immediately by confocal microscopy (LSM 710, Carl Zeiss, Göttingen, Germany). Emission was collected at 530 ± 20 nm (annexin V) and 620 ± 20 nm (propidium iodide) upon excitation at 514 nm.

1.13 Measurement of Caspase 3/7 Activity and Intracellular ATP Levels Assays

Measurement of caspase 3/7 activity was determined using Caspase-Glo 3/7 kit (Promega) and adenosine triphosphate (ATP) content was carried out using the CellTiter-Glo kit (Promega) according to the manufacturer's instructions. Briefly, the cells were seeded in 96-well plates, cultured for 24 h, and then treated with **LysoIr@PDA-CD-RGD** for 12 h. The cells were irradiated with an 808 nm laser (1 W cm^{-2}, 5 min). Relative luminescent units (RLU) were detected with a microplate reader (Infinite F200, Tecan, Switzerland). The results are averaged among 3 replicates and have been normalized by the values obtained on untreated control cells. Error bars represent the standard deviation.

1.14 Western Blot Analysis

U87 cells cultured in 10 cm tissue culture dishes (Corning) were treated with **LysoIr@PDA-CD-RGD** (20 μg mL^{-1}) for 12 h. The combined group was irradiation by an 808 nm laser for 5 min. After a further incubation for 12 h, the cells were

harvested. For the positive control, the cells were treated with cisplatin (50 μM, 24 h). The cell lysates were prepared in radioimmunoprecipitation assay (RIPA) buffer supplemented with inhibitors of proteases (Roche Diagnostics GmbH, Germany) and inhibitor of phosphatases sodium orthovanadate (Sigma Aldrich). Protein concentrations were quantified by a BCA protein assay reagent kit (Novagen Inc, USA). Equal amounts of protein were loaded and separated on SDS-polyacrylamide gel electrophoresis and then transferred onto polyvinylidene difluoride membranes (Millipore, USA). The membranes were blocked with 5% non-fat dry milk in TBST (20 mM Tris/HCl (pH 7.2)/150 mM NaCl/0.05% Tween-20) at room temperature for 2 h, and subsequently incubated with primary antibodies specific to β-actin (Cell Signaling Technology, USA), caspase-3 (Cell Signaling Technology, USA) in TBST containing 5% nonfat milk at 4 °C. The membranes were then washed with TBST for three times and incubated with HRP-conjugated secondary antibody for 1 h at room temperature. After the samples were washed at least three times with TBST, the signals were detected using the enhanced chemiluminescence (ECL) kit (Amersham Inc, USA). Images were captured on FluorChem M (ProteinSimple, Santa Clara, CA).

1.15 Detection of Intracellular ROS

After treated with **LysoIr@PDA-CD-RGD** (20 μg mL^{-1}) for 12 h, the cells were irradiated with an 808 nm laser (1 W cm^{-2}, 10 min). The cells were then incubated with DCFH-DA (10 μM) in serum-free medium for 15 min at 37 °C in the dark. After washed twice with serum-free DMEM, the samples were analyzed by confocal microscopy with excitation at 488 nm and emission at 530 ± 15 nm.

1.16 Detection of Cathepsin B Release

Cathepsin B activity was detected using the fluorogenic substrate Magic Red MR-$(RR)_2$ according to the manufacturer's instructions. Briefly, U87 cells seeded into 35 mm dishes (Corning) were treated with **LysoIr@PDA-CD-RGD** (20 μg mL^{-1}) for 24 h. For PTT treatment, cells were irradiated with an 808 nm laser (1 W cm^{-2}, 5 or 10 min). The cells were washed twice with PBS and then incubated with Magic Red MR-$(RR)_2$ at 37 °C for 1 h. After washed twice with PBS, the cells were visualized by confocal microscopy. Emission was collected at 630 ± 20 nm upon excitation at 543 nm.

1.17 Detection of lysosomal pH

U87 cells was treated with **LysoIr@PDA-CD-RGD** (20 μg mL^{-1}) for 4 h and stained with Lyso-DR (1 μg mL^{-1}) for 30 min. One of group was irradiated by an 808 laser for (1 W cm^{-2}, 5 min). The other groups were kept in dark. Then, the U87 cells were observed under a confocal microscope after 30 and 60 min. Dansyl: λ_{ex} = 405 nm; λ_{em} = 440 ± 30 nm. R6G-amide: λ_{ex} = 543 nm; λ_{em} = 590 ± 30 nm.

1.18 Two-photon Phosphorescence Lifetime Imaging

U87 cells were treated with **LysoIr** (2 μM**,** 1 h) and **LysoIr@PDA-CD-RGD** (20 μg mL^{-1}, 2 h). The media was removed and the cells were then incubated with nigericin (20 μM) in disodium hydrogen phosphate/citric acid buffer solutions for 10 min. Two-photon excited, time-resolved phosphorescence lifetime imaging of U87 cells was conducted by using a TI: SAPPHIRE pulsed laser (Coherent Chameleon) on Zeiss LSM 710 confocal microscope combined with a Becher & Hickl (bh)

time-correlated single photon counting (TCSPC) system. PLIM data was processed using SPCImage software (Becker & Hickl GmbH, the bh TCSPC Handbook sixth Edition. Available on www.becker-hickl.com). Two-photon excitation of Ir(III) complex was performed at 810 nm and with an image format of 512 × 512.

1.19 Animals

Female BALB/c nude mice were obtained from Beijing Vitalriver Experimental Animal Technology Co. Ltd. with body weights of 19~21 g and housed in stainless steel cages under the standard conditions (20 ± 2 ^{o}C room temperature, 60 ± 10% relative humidity) with a 12 h light/dark cycle. Distilled water and sterilized food for mice were available ad libitum. Animals were acclimated to this environment for 5 days prior to treatment. All procedures used in this experiment were compliant with the local animal ethics committee.

1.20 *In Vivo* Photothermal and Photoacoustic Imaging

U87-tumor-bearing mice were intratumorally (i.t) or intravenously (i.v.) injected with **LysoIr@PDA-CD-RGD** (2 mg mL^{-1}, 100 μL) for PA imaging. Thermal imaging was recorded by a thermal camera (MAG30, Magnity Electronics, Thermal Imaging Expert) when the tumors were exposed to an 808 nm laser with a power density (i.t. injection group : 1 W cm^{-2}, Control and i.v. injection group : 1.5 W cm^{-2}). for 10 min. PA imaging was conducted using a Vero LAIR animal photoacoustic and ultrasound imaging system (Nexus128, USA).

1.21 *In Vivo* Biodistribution Measurement of LysoIr@PDA-CD-RGD

Mice bearing U87 tumors were i.v. injected with **LysoIr@PDA-CD-RGD** (2 mg mL^{-1}, 200 μL) and sacrificed at 1, 2, 4 and 7 day post injection. The major organs (heart, liver, spleen, lung, kidney and tumor) were collected, wet weighed and dissolved in aqua regia. The amount of acid added was at least ten times as the weight of each tissue sample. The samples were heated at 200 °C for 0.5 h. After cooling down to room temperature, each of resulting solutions was then diluted by water to 10 ml, and subsequently analyzed by ICP-MS to determine the total amount of Ir in each measured organ. Serial dilutions of standard Ir samples were used for calibrations. Three animals per group were used in the biodistribution measurement. The Ir levels in organs were presented in the unit of the percentage of injected dose per gram tissue (%ID/g).

1.22 *In Vivo* Anticancer Properties

When tumor volumes reached 100~150 mm^3, U87-tumor-bearing mice were divided into the following six groups (five mice per group): (1) Control; (2) NIR (1.5 W cm^{-2}); (3) **LysoIr@PDA-CD-RGD**: Intratumoral injection (4) **LysoIr@PDA-CD-RGD**: Intravenous injection (5) **LysoIr@PDA-CD-RGD**: Intratumoral injection + NIR (6) **LysoIr@PDA-CD-RGD**: Intravenous injection + NIR. Mice in Groups 3, 4, 5 and 6 were injected with **LysoIr@PDA-CD-RGD** (200 μL, 2 mg mL^{-1}), and mice in groups 1 and 2 were intratumoral injected with saline. After 4 h, mice in groups 5 was exposed to an 808 nm laser (1 W cm^{-2}) for 10 min and group 2 and 6 was exposed to an 808 nm laser (1.5 W cm^{-2}) for 10 min; The volumes of tumor were measured at day 14 after various treatments using digital

caliper every 2 days. The volume of tumor = length × width^2/2. Tumors and major organs (heart, liver, spleen, lung, and kidney) of mice in all groups were dissected 14 d after treatment for paraffin section and staining by H&E. Finally, the tissue sections were observed by digital microscope (Leica QWin).

1.23 Statistical Analysis

All biological experiments were performed at least twice with triplicates in each experiment. Representative results were depicted in this report and data were presented as means ± standard deviations (SD).

2. Supporting Figures and Tables

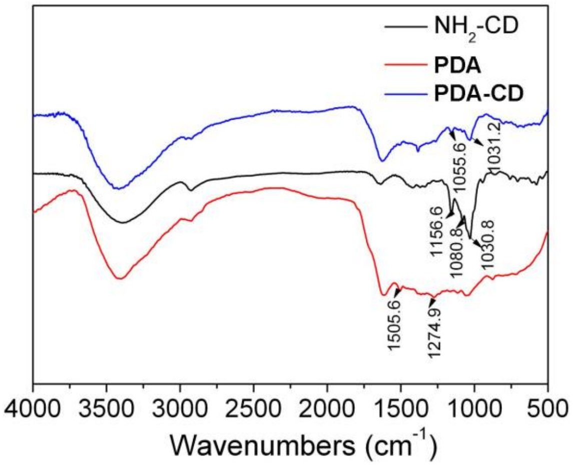

Figure S1. The FT-IR spectra of **PDA**, **PDA-CD** and NH_2-CD.

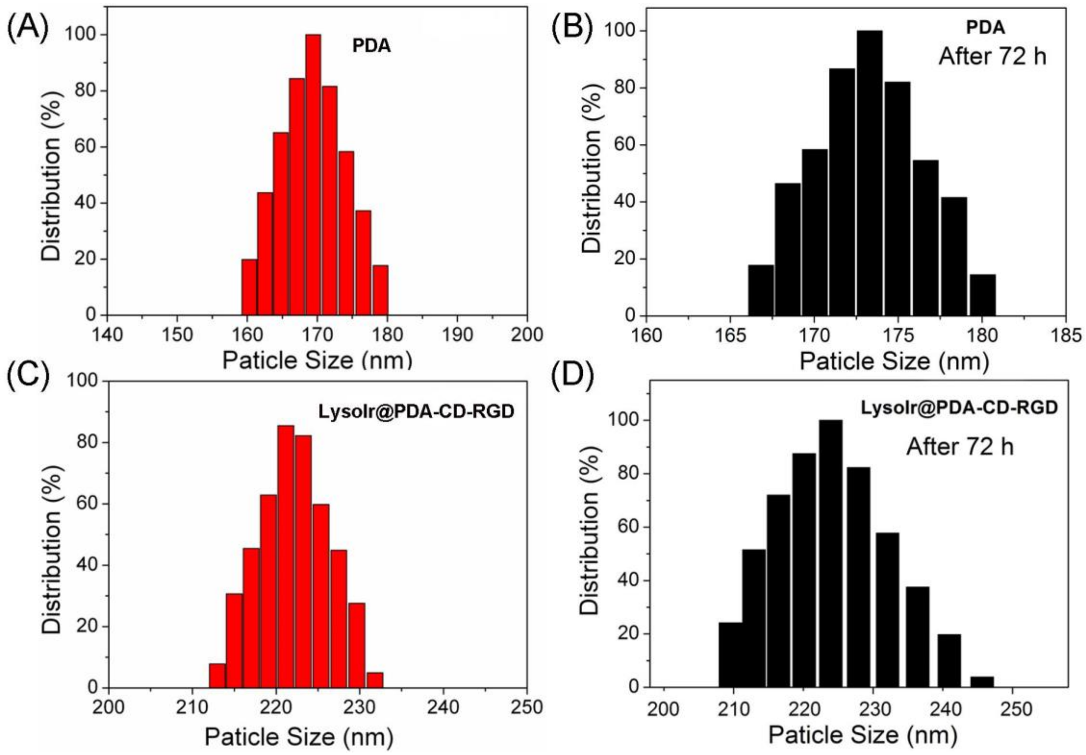

Figure S2. Number-average diameter of **PDA** (A) and **LysoIr@PDA-CD-RGD** (B) in water, measured by DLS. Number-average diameter of **PDA** (C) and **LysoIr@PDA-CD-RGD** (D) measured by DLS in water after 72 h.

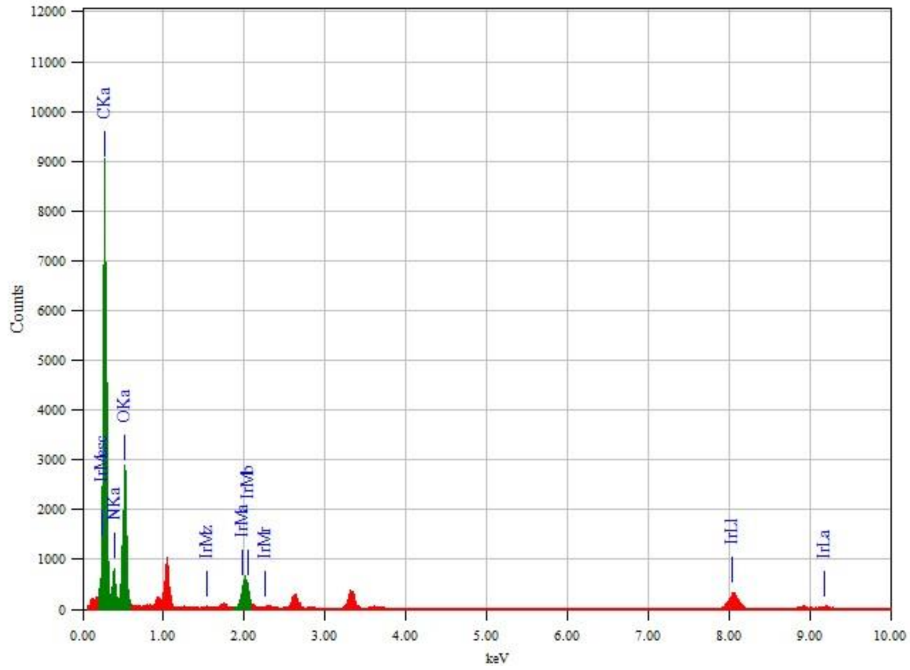

Figure S3. The EDX spectrum of as-synthesized **LysoIr@PDA-CD-RGD** nanoparticles.

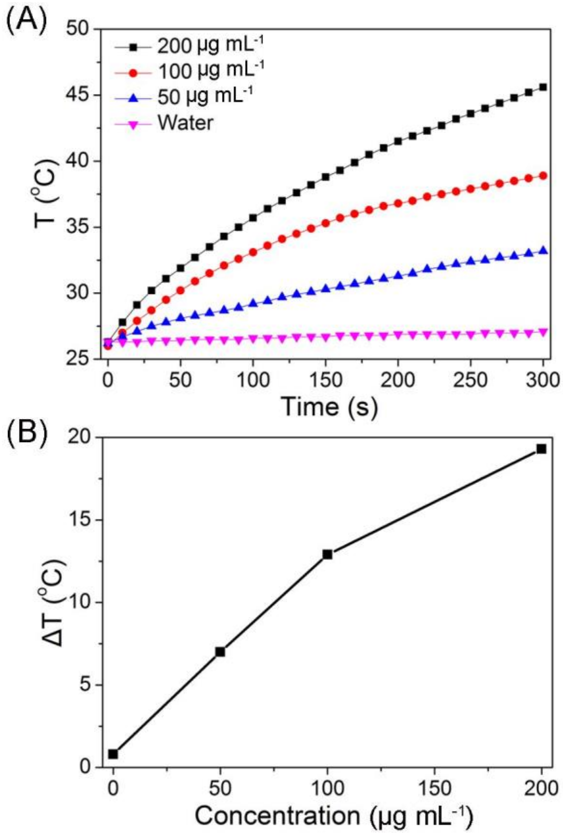

Figure S4. (A) The photothermal profiles of pure water and aqueous dispersions of **PDA** NPs at different concentrations under 808 nm laser irradiation at a power density of 1 W cm^{-2}. (B) Plot of temperature change (ΔT) of **PDA** NPs solution at different concentrations over a period of 5 min.

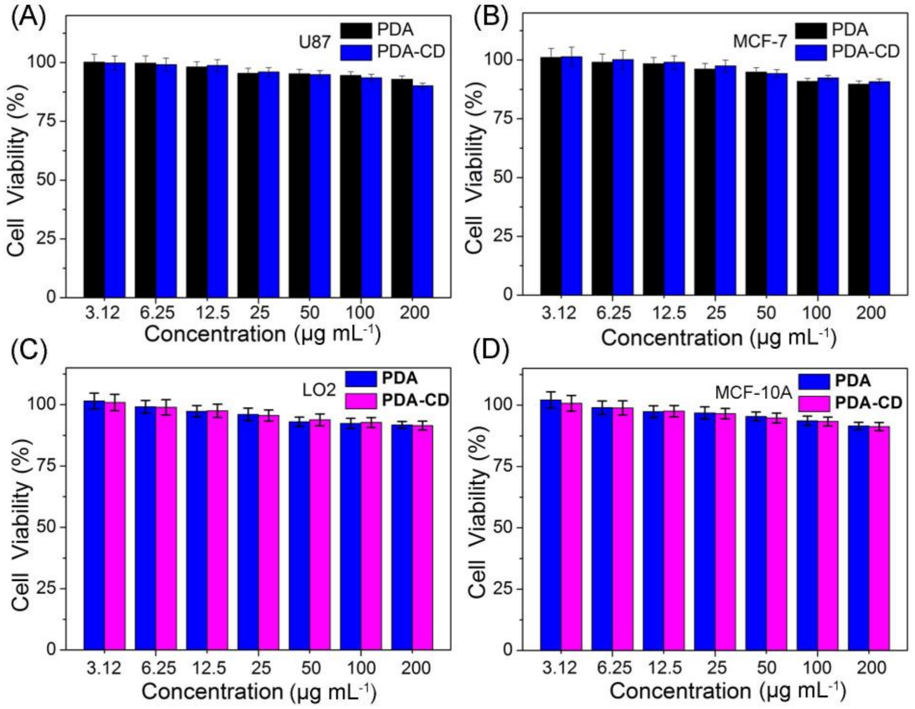

Figure S5. Cytotoxicity of the **PDA** and **PDA-CD** towards U87, MCF-7 cells, LO2 and MCF-10A cells.

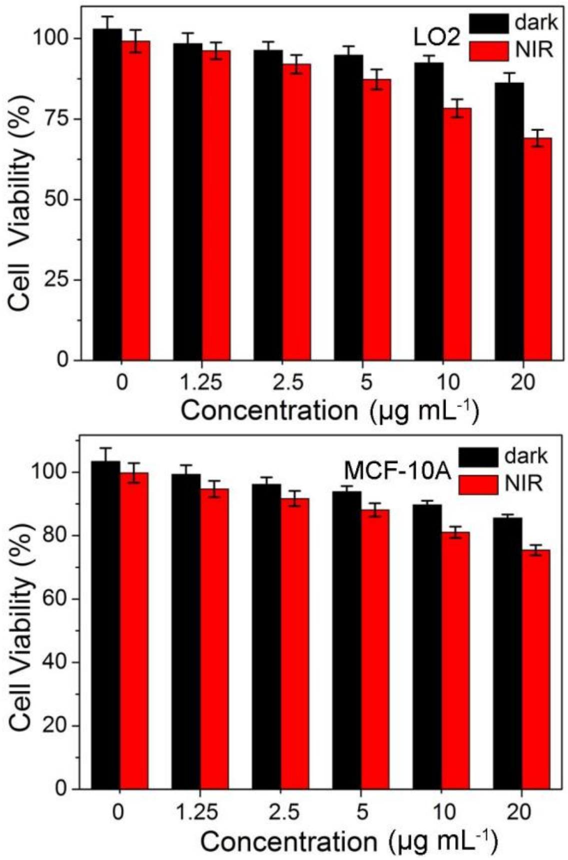

Figure S6. Cytotoxicity of the **LysoIr@PDA-CD-RGD** towards LO2, MCF-10A cells.

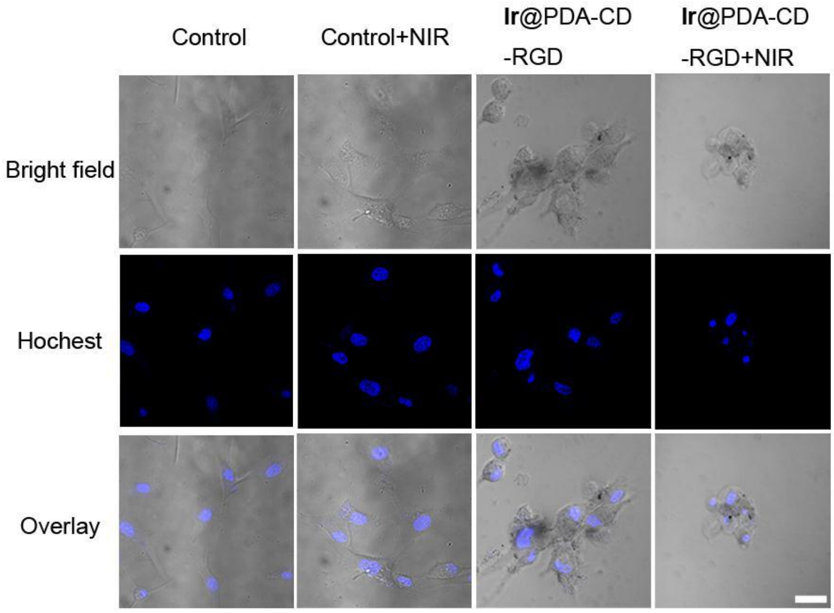

Figure S7. Hoechst 33342 stained U87 cells after the cells were treated with **LysoIr@PDA-CD-RGD** (20 μg mL^{-1}, 12 h) and irradiated with the 808 nm laser (1 W cm^{-2}) or not. Scale bar: 10 μm.

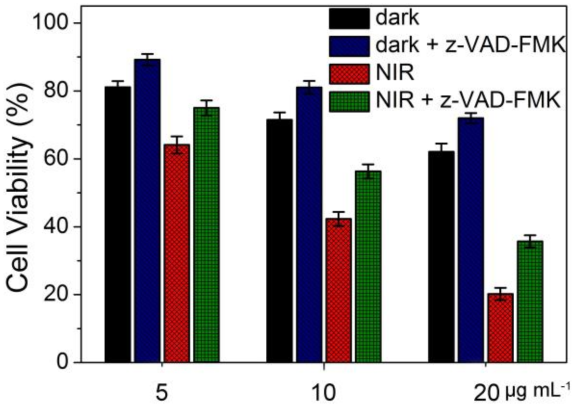

Figure S8. U87 cells were treated with **LysoIr@PDA-CD-RGD** at the indicated concentrations in the absence or presence of z-VAD-FMK. Cells viability was measured by MTT assay. The cells were irradiated with an 808 nm laser at a light dose of 1 W cm^{-2} for 10 min.

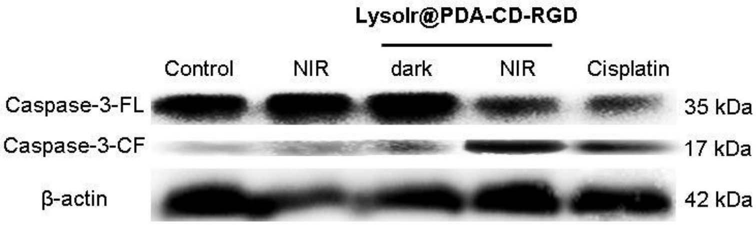

Figure S9. U87 cells were treated with **LysoIr@PDA-CD-RGD** (20 μg mL^{-1}) for 24 h. The full-length and cleaved protein fragments of caspase-3 were detected by Western blot (FL: full length; CF: cleaved form).

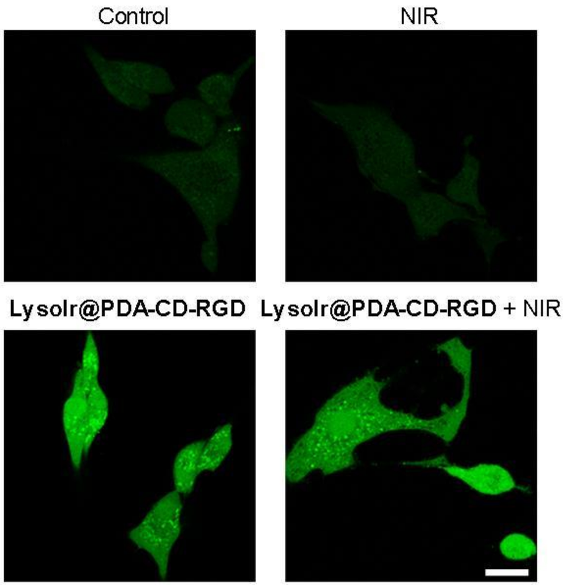

Figure S10. Confocal microscopic images of cellular ROS levels detected by DCFH-DA staining of U87 cells treated with **LysoIr@PDA-CD-RGD** in the absence or presence of light. DCF: λ_{ex} = 488 nm; λ_{em} = 530 ± 20 nm. Scale bar: 10 μm.

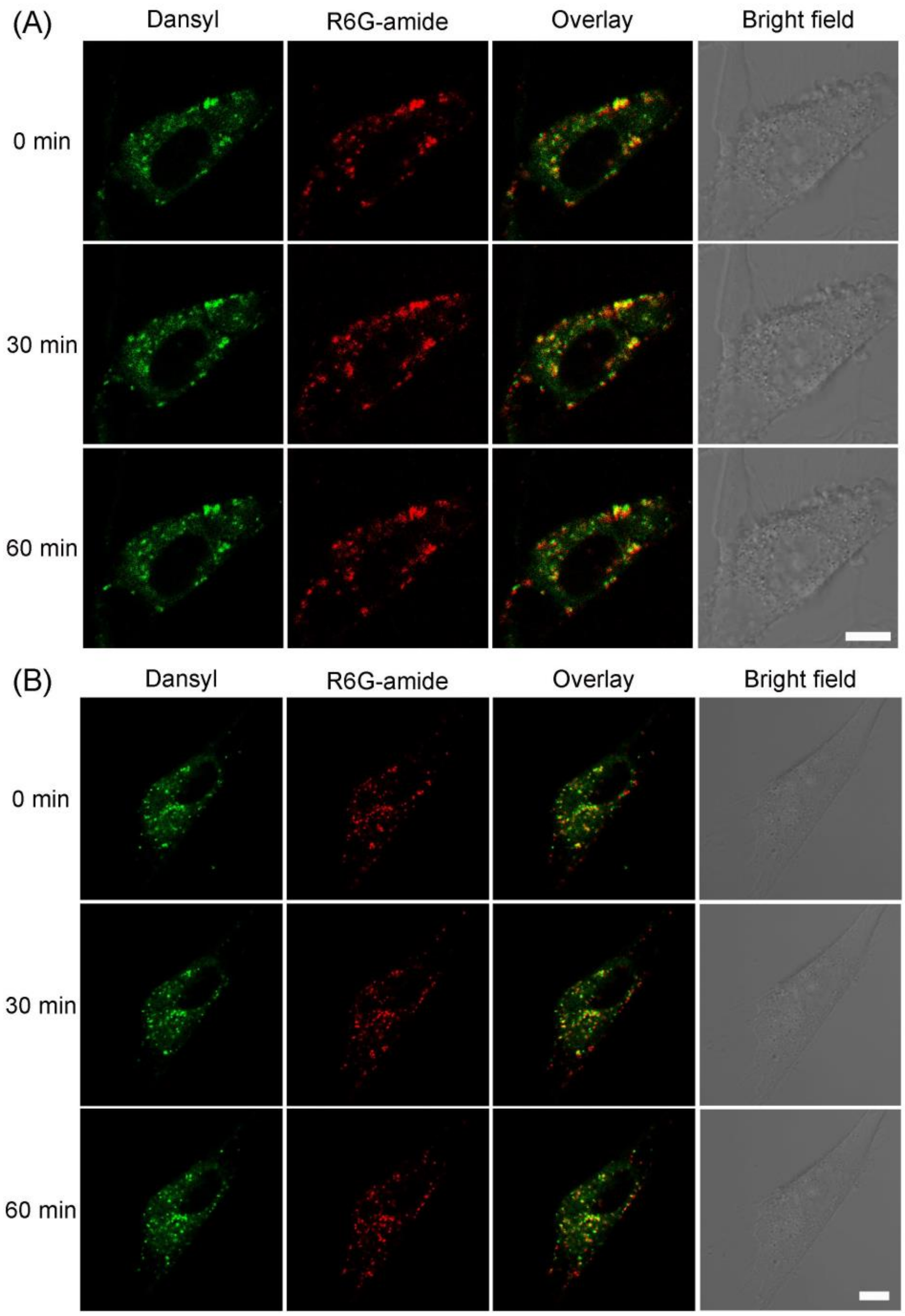

Figure S11. Visualization of lysosomal pH in a U87 cell. U87 cells was incubated in DMEM medium supplemented Lyso-DR (1 μg mL^{-1}) for 30 min and probed at 0, 30 and 60 min by confocal fluorescence microscopy. (A) is the control group and (B) is treated with **LysoIr@PDA-CD-RGD** (20 μg mL^{-1}) for 4 h in the dark. Dansyl fluorescence was shown in green (λ_{ex} = 405 nm; λ_{em} = 440 ± 30 nm) and R6G-amide fluorescence was shown in red (λ_{ex} = 543 nm; λ_{em} = 590 ± 30 nm). Scale bars: 10 μm.

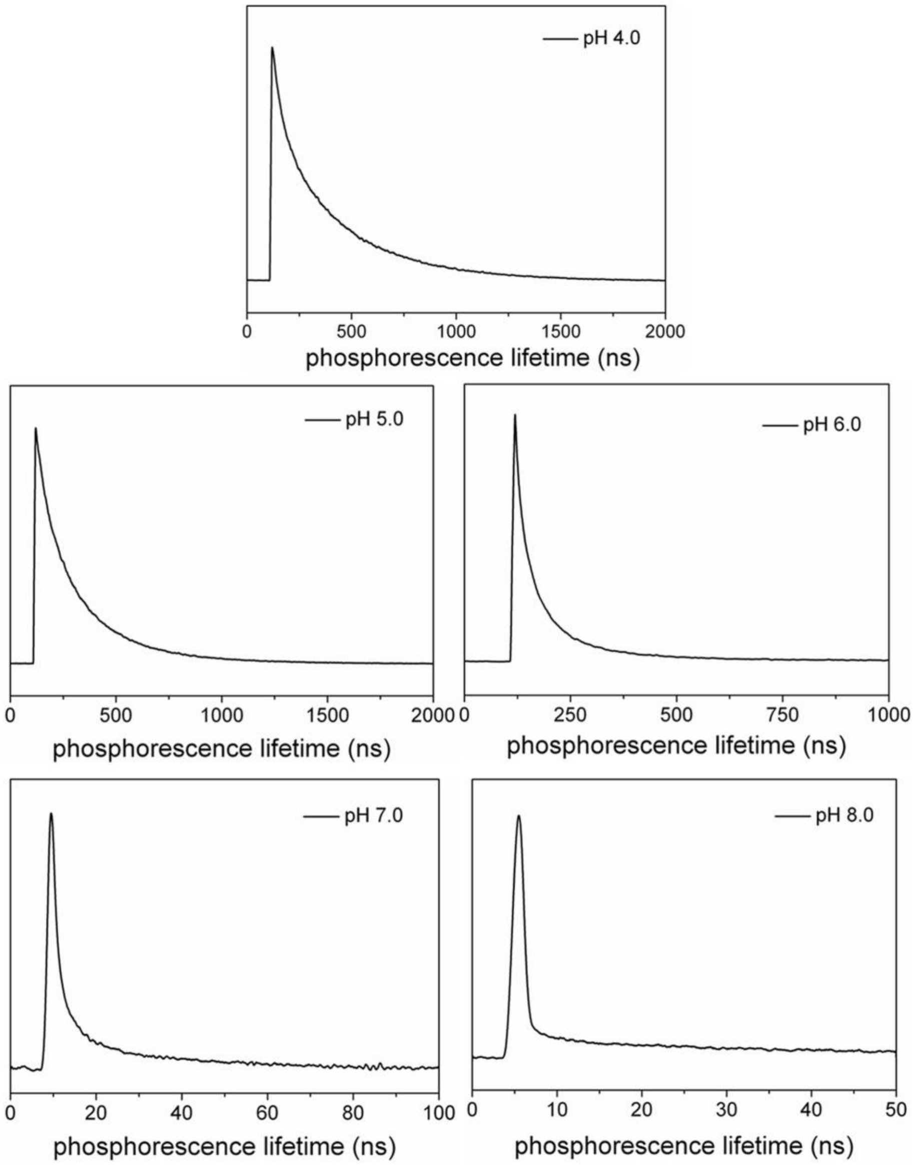

Figure S12. pH-sensitive phosphorescence lifetime of **LysoIr** (10 μM, 405 nm) in disodium hydrogen phosphate/citric acid buffer solutions.

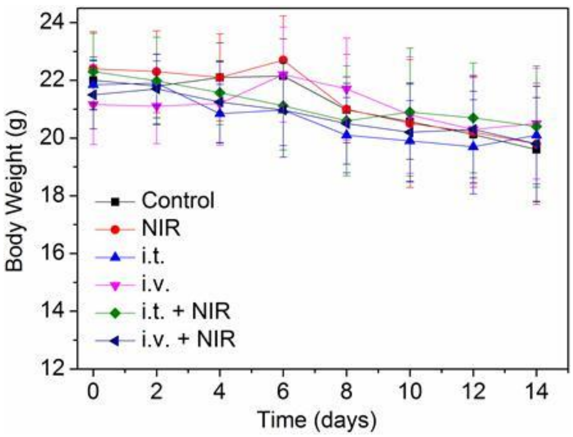

Figure S13. Body weight data of mice after various treatments indicated.

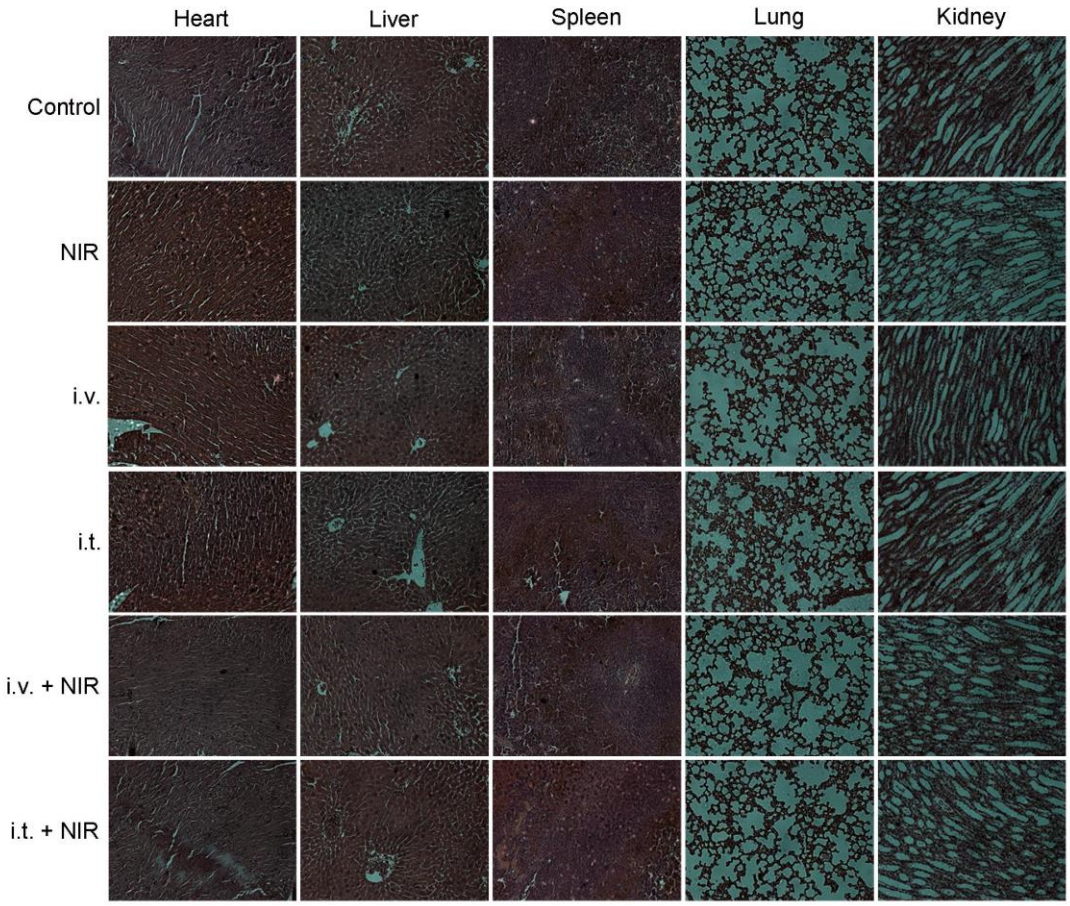

Figure S14. H&E stained slices of major organs of healthy mice and mice 14 d post-**LysoIr@PDA-CD-RGD** i.v. and i.t. injection.

Table S1 pH-sensitive phosphorescence lifetime of **LysoIr** in disodium hydrogen phosphate/citric acid buffer solutions

pH	4.0	5.0	6.0	7.0	8.0
Lifetime (ns)	340.5	249.5	157.6	12.2	1.3

References

1. L. He, C. P. Tan, R. R. Ye, Y. Z. Zhao, Y. H. Liu, Q. Zhao, L. N. Ji, Z. W. Mao, *Angew. Chem. Int. Ed.* **2014**, *53*, 12137.
2. D. Zhang, M. Wu, Y. Zeng, L. Wu, Q. Wang, X. Han, X. Liu, J. Liu, *ACS Appl. Mater. Interfaces* **2015**, *7*, 8176.
